# Hysteresis of haptic vertical and straight ahead in healthy human subjects

**DOI:** 10.1186/1471-2202-13-114

**Published:** 2012-09-22

**Authors:** Alexander A Tarnutzer, Jeanine R Schuler, Christopher J Bockisch, Dominik Straumann

**Affiliations:** 1 Department of Neurology, University Hospital Zurich, Frauenklinikstrasse 26, CH-8091, Zurich, Switzerland; 2 Department of Otorhinolaryngology, University Hospital Zurich, Zurich, Switzerland; 3 Department of Ophthalmology, University Hospital Zurich, Zurich, Switzerland

**Keywords:** Perception, Pointing, Pseudo-neglect, Somatosensory, Kinesthetics

## Abstract

**Background:**

The subjective haptic vertical (SHV) task requires subjects to adjust the roll orientation of an object, mostly in the roll plane, in such a way that it is parallel to perceived direction of gravity. Previously we found a tendency for clockwise rod rotations to deviate counter-clockwise and vice versa, indicating hysteresis. However, the contributing factors remained unclear. To clarify this we characterized the SHV in terms of handedness, hand used, direction of hand rotation, type of grasping (wrap vs. precision grip) and gender, and compared findings with perceived straight-ahead (PSA). Healthy subjects repetitively performed adjustments along SHV (n = 21) and PSA (n = 10) in complete darkness.

**Results:**

For both SHV and PSA significant effects of the hand used and the direction of rod/plate rotation were found. The latter effect was similar for SHV and PSA, leading to significantly larger counter-clockwise shifts (relative to true earth-vertical and objective straight-ahead) for clockwise rotations compared to counter-clockwise rotations irrespective of the handedness and the type of grip. The effect of hand used, however, was opposite in the two tasks: while the SHV showed a counter-clockwise bias when the right hand was used and no bias for the left hand, in the PSA a counter-clockwise bias was obtained for the left hand without a bias for the right hand. No effects of grip and handedness (studied for SHV only) on accuracy were observed, however, SHV precision was significantly (p < 0.005) better in right-handed subjects compared to left-handed subjects and in male subjects.

**Conclusions:**

Unimanual haptic tasks require control for the hand used and the type of grip as these factors significantly affect task performance. Furthermore, aligning objects with the SHV and PSA resulted in systematic direction-dependent deviations that could not be attributed to handedness, the hand used, or the type of grip. These deviations are consistent with hysteresis and are likely not related to gravitational pull, as they were observed in both planes tested, i.e. parallel and perpendicular to gravity. Short-term adaptation that shifts attention towards previous adjustment positions may provide an explanation for such biases of spatial orientation in both the horizontal and frontal plane.

## Background

Accurate and precise estimates of the body’s orientation relative to the direction of gravity are crucial in daily life to maintain balance and to interact with the environment. Multimodal sensory input, originating from the vestibular organs (saccular and utricular macula, semi-circular canals), proprioceptors (located in the skin, muscles and joints) and vision is integrated by the central nervous system to continuously update an internal estimate of the direction of gravity [1]. Verticality perception can be assessed in three different modalities: the visual vertical, the haptic vertical and the postural vertical. Each of these three modalities has its advantages and disadvantages and may be more or less suitable depending on the specific aims of the study and the characteristics of the participants. Sensory signals might be weighted differently in these modalities. For example, the visual vertical is sensitive to vestibular nuclei lesions, while the haptic vertical remains within the normal range, indicating dissociation between these two modalities [2]. Patients with postural imbalance should preferentially be assessed using the postural vertical and the haptic vertical, as these two modalities were found to be more closely related to postural disorders than the visual vertical [3]. The majority of studies addressing verticality perception, however, focuses on vision-based measurements by asking subjects to rotate a luminous line in the roll plane in otherwise complete darkness to an orientation parallel to the perceived vertical, a task termed “subjective visual vertical” (SVV). In upright head orientation healthy human subjects accurately adjust the SVV within ±2.5° of true earth-vertical [3,4]. The subjective haptic vertical (SHV), on the other hand, requires interaction with the environment by grasping, holding and manipulating objects in complete darkness [5], strongly relying on tactile and kinesthetic input (see [6] for a review of the haptic perception of spatial orientations). In upright position, adjustment errors of the SVH up to ±4° (for bimanual adjustments [2]) and ±4.5° (for unimanual adjustments [3]) of true earth-vertical are considered normal. Whereas both over- and undershooting of the hand during reaching movements to remembered targets in three-dimensional space [7,8] were observed, less is known about errors of haptic tasks in the roll plane. In previous publications deviations of the perceived earth-horizontal and earth-vertical in haptic roll alignment tasks with subjects being in an upright position were found to vary substantially. In a study by Wade and Curthoys [9], subjects were accurate when bimanually adjusting a rod to perceived earth-horizontal in complete darkness. In contrast, Lejeune and coworkers [10] found systematic undershooting of adjustments when tactile input was restricted to slight touch of the movable rod. The different results of these studies suggest that the mode of tactile information influences haptic task performance. Apart from the type of grip used, unimanual adjustments seem to depend on the hand used, as shown by Bauermeister and colleagues [11].

**Figure 1:**
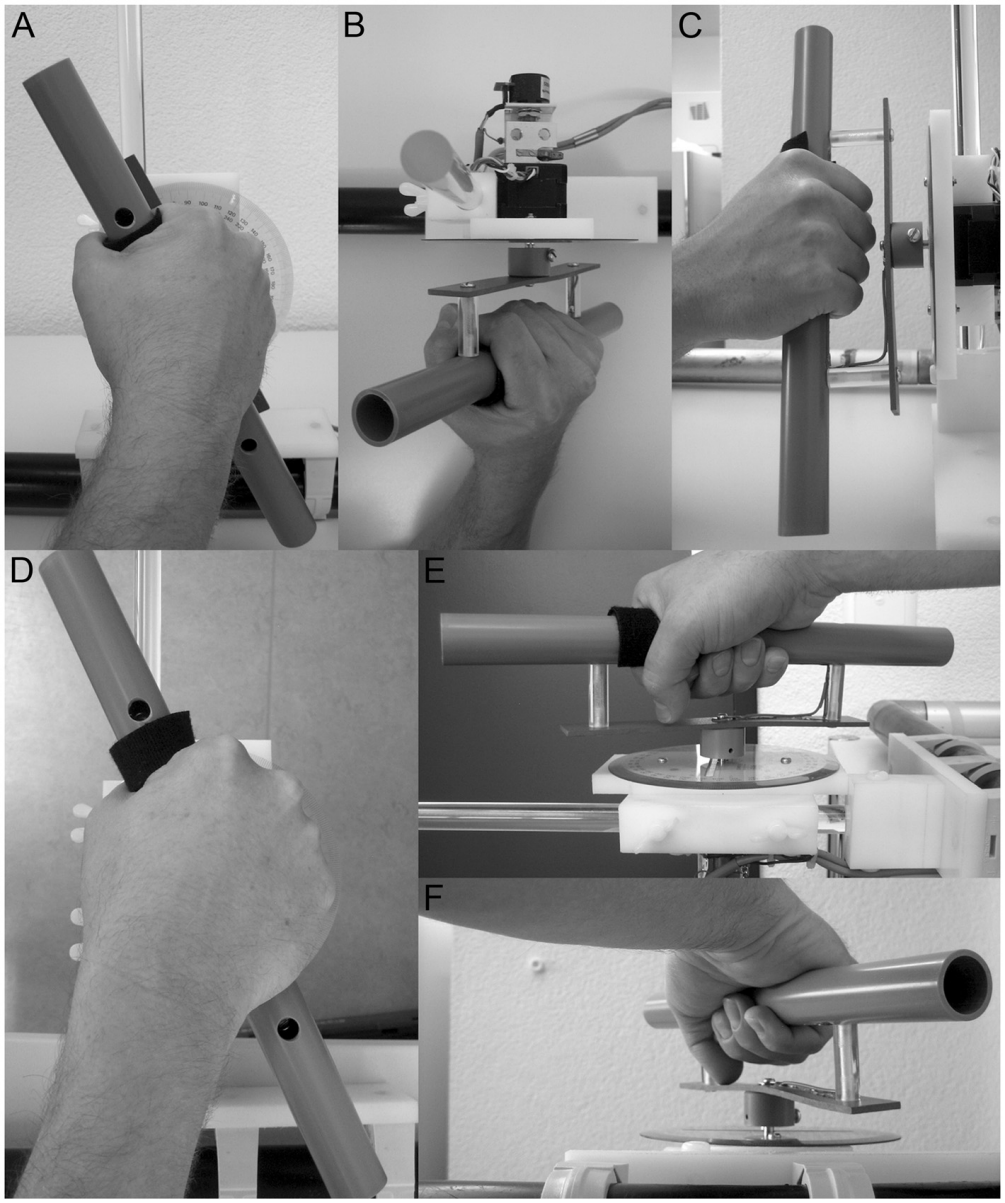
**Photographs in the three principle planes (frontal, sagittal, and axial) for both the SHV (panels A-C) and the PSA (panels D-F) task when holding the tactile bar that was positioned in front of the subject in the median sagittal plane.** Rotations in the frontal plane for the SHV (panel **A**, frontal view) correspond to rotations in the axial plane for the PSA (panel **D**, top view). The view from top for the SHV (panel **B**, axial plane) matches the frontal view for the PSA (panel **F**, frontal plane) and the view from the side for the SHV (panel **C**, sagittal plane) parallels the side view for the PSA (panel **E**, sagittal plane).

Pointing straight-ahead is used to indicate perceived body midline (also termed “perceived straight-ahead” or PSA). The body midline, in turn, divides the body and space in two equal left and right parts [12]. Healthy human subjects systematically mis-bisect space when pointing straight-ahead by a few degrees [13,14]. However, considerable between-study variability characterizes the literature as noted in a meta-analysis [15]. In particular, the direction of the movement from the starting position (from left-to-right vs. from right-to-left) and the hand used were found to significantly affect adjustments of PSA in this meta-analysis. The PSA can experimentally be transformed to a haptic task by letting subjects point an object (e.g. a rod) along the perceived straight-ahead direction. From the motor output perspective, the left-right orientation of the rod in PSA (Figure 1D-top view of PSA) corresponds to the torsional orientation of the rod in the SHV task (Figure 1A-frontal view of SHV). However, only in the SHV gravity perception will influence the setting in a meaningful way. Figure 1 depicts the similarities and differences between SHV and PSA with a rod. As noted, the extent of flexion/extension in the wrist and the elbow required to solve the SHV and the PSA task may be different, potentially leading to distinct activation of joint receptors. 

However, if the pattern of adjustment errors in SHV and PSA are mainly related to motor factors and not to the orientation of gravity, we expect similar error patterns in both planes. A clockwise (CW) deviation of SHV (as from the subject’s perspective), for example, is proposed to translate to a rightward deviation of PSA if motor factors dominate. However, based on previous studies, additional factors apart from rod orientation (SHV vs. PSA) and potentially distinct wrist/elbow joint receptor stimulation may play a role in the performance of subjects also. These include 1) the type of grip, 2) handedness, 3) the hand used, and 4) the direction of rotation of the haptic device:

1) As noted by others the type of grip may affect haptic adjustments in the roll plane [9,10]. We therefore studied SHV and PSA adjustments using two distinct types of grasp. Whereas grasping a rod will lead to firm touch of the entire palm yielding large area tactile input (= wrap grip), a small plate will restrict touch to the fingertips (= precision grip) resembling more a kinesthetic task as the one used by Lejeune and colleagues [10]. At the same time, use of the fingertips increases the number of joints involved and consequently allows more degrees of freedom. We hypothesize that by performing the task with the fingertips the precision of adjustments decreases, as additional degrees of freedom of joint movements will add noise.

2) It has been proposed that during aimed movements the dominant arm shows advantages for coordinating inter-segmental dynamics as for specifying trajectory speed and direction [16,17], while the non-dominant arm shows advantages in controlling limb impedance, as required for accurate final position control [17,18]. Studying reaching movements, each hemisphere/limb system appeared specialized for stabilizing different aspects of task performance [19]. Whereas asymmetries in favor of the preferred arm were found in studies assessing the role of visual information on targeted movement [20-22], a non-preferred arm advantage in the ability to utilize proprioceptive feedback was suggested [23-26]. However, for haptic tasks in the roll plane, this hypothesis has not been evaluated. We will therefore compare performance in right- and left-handed subjects using both the dominant and the non-dominant hand and evaluate whether accuracy is indeed higher for the non-dominant hand as suggested in the literature. We will also compare the precision of adjustments in these subjects.

3) With regards to unimanual haptic tasks, previous work suggests an effect of the hand used on adjustment errors for the SHV with left-handed adjustments deviating CW and right-handed adjustments deviating counter-clockwise (CCW) [11], while for the PSA to our knowledge this has not been studied in detail.

4) There is evidence that the direction of rotation (CW vs. CCW) of a tactile device has a significant impact on the adjustment error in the roll plane. Such direction-dependent differences are referred to as hysteresis, i.e., indicate that the perceived position of the hand depends upon the recent history of hand positions [27]. More specifically, hysteresis is a lagging or retardation of the effect, when the forces acting on a body are changed (Merriam Webster definition). Whether these direction-dependent effects resemble those found in the PSA as noted by [15], however, has not been assessed. Based on the shared features of the two tasks we would predict a similar pattern of hysteresis in the PSA compared to the SHV. However, both the differences in the plane of action relative to gravity and the differences in wrist and elbow flexion may result in partially distinct patterns.

## Methods

Two experimental setups are described here. Experiment 1 addressed verticality perception in the roll plane by means of the subjective haptic vertical (SHV), and experiment 2 focused on perceived straight ahead (PSA) in the horizontal plane.

### Subjects

Twenty-one healthy human subjects (12 women, 9 men), aged between 23 and 43 years participated in experiment 1. Two participants were familiar with the experimental setting, 19 subjects were naïve. Handedness was determined using a 13-item questionnaire [28]. Twelve subjects (7 female, 5 male) were right-handed, nine subjects (5 female, 4 male) were left-handed. Ten healthy, right-handed human subjects (5 women, 5 men; 24–44 years old) participated in experiment 2. Five of these subjects had already participated in experiment 1. We restricted experiment 2 to right-handed subjects, as we were mainly interested in the accuracy of adjustments, where findings from experiment 1 suggested no principle differences between right- and left-handed subjects.

Informed consent of all subjects was obtained after full explanation of the experimental procedure. The protocol was approved by the Ethics Committee Neurology of the University Hospital Zurich, and was in accordance with the ethical standards laid down in the 1964 Declaration of Helsinki for research involving human subjects.

### Experimental setting

**Figure 2:**
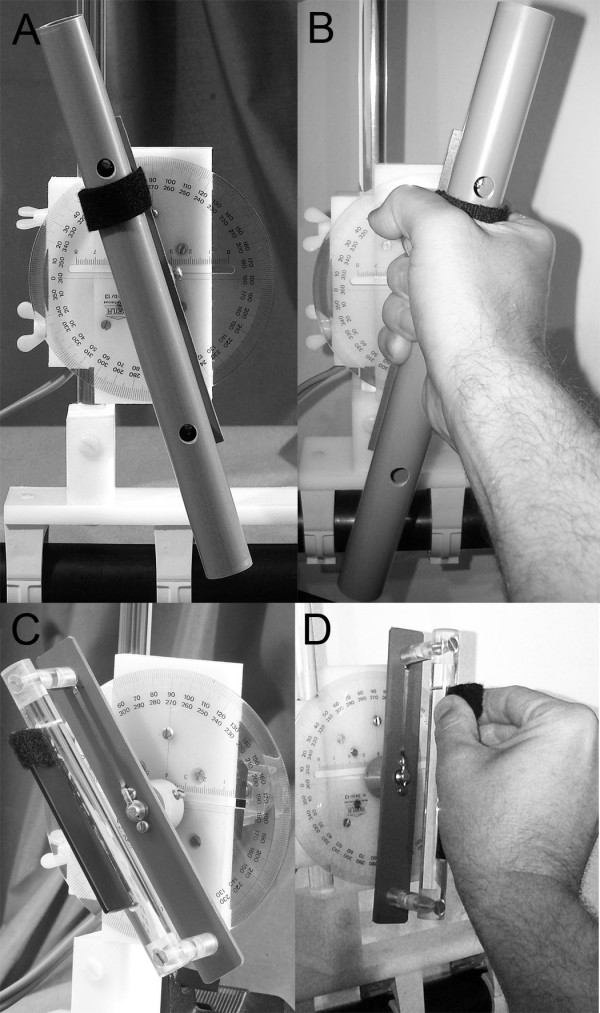
**Photographs of the two tactile devices (panel A: tactile rod; panel C: tactile plate) used in experiment 1 and 2.** For the tactile rod, firm grasp using a wrap grip with the entire palm is mandatory (panel **B**), whereas for the tactile plate slight touch with the fingertips is needed (panel **D**). Grasping either device was done in such a way that the Velcro is located between the thumb and the index finger, as shown in Figure 2B (Velcro partially covered by the grasping hand) and Figure 2D.

Subjects were seated in upright position. While viewing straight-ahead a thermoplastic mask (Sinmed BV, Reeuwijk, The Netherlands) that tightly covered the head was applied and attached to a base plate behind the subject’s head. Vacuum pillows were placed on both sides of the hips enabling subjects to support their elbows there during the experiment. Two tactile devices were used to assess perceived vertical (see Figure 2), demanding either a medium wrap grip (tube, 29 cm long, 2.5 cm thick: large areas of contact between the object and the fingers and palm, with little or no ability to impart motions with the fingers; Figure 2B) or a precision grip of a rectangular plate (dimensions: 87 mm x 10 mm x 1 mm, small areas of contact between the object and the fingers, motions mainly with the fingers; Figure 2D). Both devices were mounted on a safety bar and placed in front of the subjects in the midline approximately 40 cm distant in the sagittal plane (experiment 1). The same two tactile devices were used in experiment 2. Both devices were positioned in the roll plane for calibration beforehand using a pendulum. For experiment 2 the devices were then pitched forward and placed in the horizontal plane along the objective body midline about 67 cm distant. By adding a Velcro strip to one end of the rod and the plate in either experiment, the two end points of the devices could be distinguished. Such a polarized stimulus has the advantage that it allows a more precise definition of the task and control of the direction of rotation.

### Experimental paradigms

For experiment 1 data collection was split into four blocks recorded in a single session, and each block contained 48 trials in pseudo-randomized order. Both adjustments using the dominant and the non-dominant hand were studied. We opted for unimanual adjustments (instead of bimanual ones as used e.g. by Wade and Curthoys [9]) as previously effects of the hand used were noted for both adjustments in the roll and the horizontal plane. We aimed to further characterize these effects in both planes.

First subjects adjusted the rod using a medium wrap grip with the dominant (block 1) and non-dominant hand (block 2). Afterwards the plate was used, requiring a precision grip, and again data collection started with the dominant hand (block 3) followed by the non-dominant hand (block 4). All experiments were run in complete darkness, i.e., the subjects had no visual feedback of their rod/plate adjustments. Subjects were instructed to grasp the device so that the Velcro strap was next to their thumb and index finger, adjust the device along the perceived vertical axis by the shortest angle of rotation possible with the Velcro up and to confirm adjustments by pushing a button with the other hand. Before data collection, subjects practiced adjustments until they could be performed reliably within six seconds. During the experiments, if the confirmation button was not pressed within this time limit, the trial was repeated later in the experiment. The starting orientation of the tactile device was 40° or 60° off earth-vertical for the SHV [deviating clockwise (CW) or counter-clockwise (CCW) as seen by the subject] or off straight-ahead (PSA, deviating left or right as seen from a top view). To reduce learning effects by repeating identical angles of rotation, a random offset within the range of ± 6° was added to each starting position. Therefore either CW or CCW rotations of the object (rod or plate) were required. A short break with the lights turned on was granted at the end of each block to allow subjects to relax and remove the mask.

### Data analysis

In both experiments, trials were sorted according to handedness (left-handed vs. right-handed), the hand used (right vs. left hand) to complete the task, the direction of rod/plate rotation (CW vs. CCW), the type of the tactile device and gender. Outliers were defined as data points differing more than two standard deviations (StdDev) from the mean. In total, 3.8% (experiment 1) and 3.4% (experiment 2) of all trials were identified as outliers and discarded. Average errors relative to earth-vertical angle (experiment 1) and relative to straight-ahead orientation (experiment 2), and StdDevs were calculated for each subject. In the following, we will use “trial-to-trial variability” whenever we report intra-individual StdDev.

If not stated otherwise, statistical analysis was done using analysis of variance (ANOVA, Minitab, Minitab Inc., State College, USA) including Tukey’s correction for multiple comparisons. Besides p-values, degrees of freedom (df) taking into consideration both the number of conditions (df_a_) and the number of participants for each condition (df_b_) are provided along the F-values. T-tests were applied to determine whether deviations in perceived vertical or straight-ahead were significant relative to true earth-vertical. Whenever multiple t-tests (ttest.m, Matlab 7, The Mathworks) were used, Holm’s correction was applied [29,30].

## Results

### Subjective haptic vertical (experiment 1)

**Figure 3:**
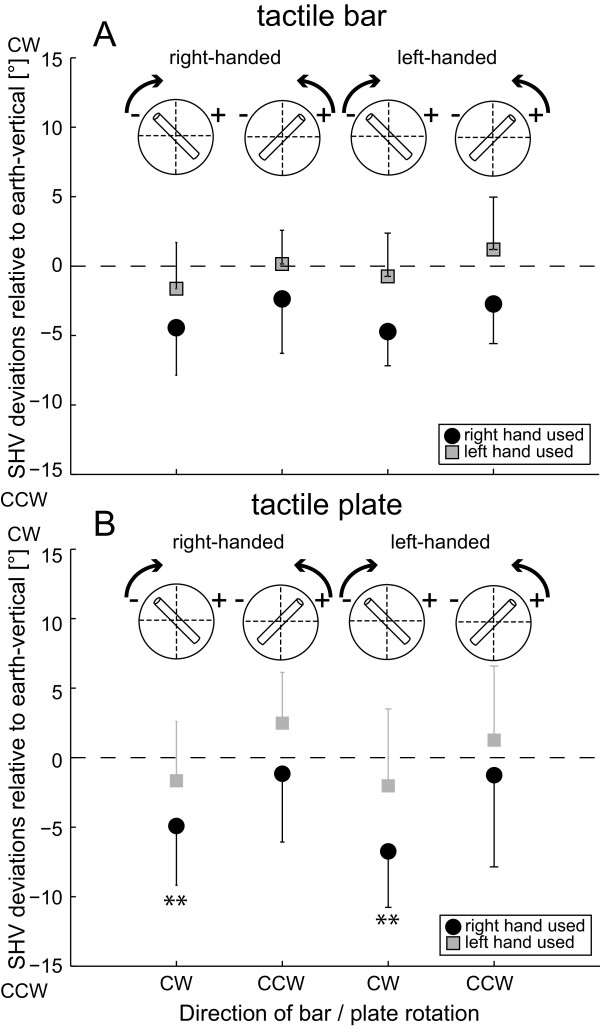
**Average (± 1 StdDev) errors of SHV (haptic vertical) for both the tactile rod (panel A) and the tactile plate (panel B) are illustrated.** Adjustments for right- and left-handed subjects are illustrated separately and errors of CW rotations are compared with those of CCW rotations. The starting position of the device and the required direction of rotation (CW vs. CCW) are also illustrated in inlets on the top. Black circles refer to adjustments with the right hand, gray squares to adjustments with the left hand. Trial types with significant errors relative to zero (t-tests) are marked (**).

Average errors of SHV relative to true earth-vertical are illustrated in Figure 3. Statistical analysis (5-way ANOVA) of SHV errors showed a significant main effect for the direction of rod/plate rotation (F[1,22] = 25.29, p < 0.001) and for the hand used (F[1,22] = 26.23, p < 0.001) while no significant main effects for handedness (F[1,22] = 0.40, p = 0.526), the type of grip used (F[1,22] = 0.15, p = 0.701) and the gender (F[1,22] = 0.03, p = 0.867) were observed. Consistently in both right- and left-handed subjects, CW rotations resulted in significantly larger CCW deviations than CCW rotations and SHV adjustments done with the right hand deviated significantly farther in the CCW direction than adjustments applied with the left hand irrespective of handedness. We found a significant interaction between the gender and the hand used (F[1,22] = 10.80, p = 0.001) and between the gender, the hand used and the type of grip (F[1,22] = 8.46, p = 0.004), while no other significant (p > 0.05) interactions between the single factors were noted.

In order to determine whether deviations in perceived vertical were significant relative to true earth-vertical, paired t-tests (comparing the actual adjustments and adjustments with a mean error of zero) were applied. When pooling all SHV trials (irrespective of the hand used, the handedness, the direction of rod/plate rotation, the type of grip and gender), significant (*t*-test, p < 0.001) deviations in the CCW direction relative to earth-vertical were found (−1.8 ± 4.6°; average ± 1 StdDev). Multiple comparisons (controlling for the factors listed above) showed that deviations were significant for right-handed subjects when rotating the object CW with the right hand to perceived vertical. This pattern was seen both for rotating the rod (−4.4 ± 3.4°, p = 0.004) and the plate (−4.9 ± 4.3°, p = 0.008). Left-handed subjects showed significant deviations when rotating the object CW with the right hand. Again, this was true for both the rod (−4.7 ± 2.4°, p = 0.002) and the plate (−6.7 ± 4.0°, p = 0.004).

**Figure 4:**
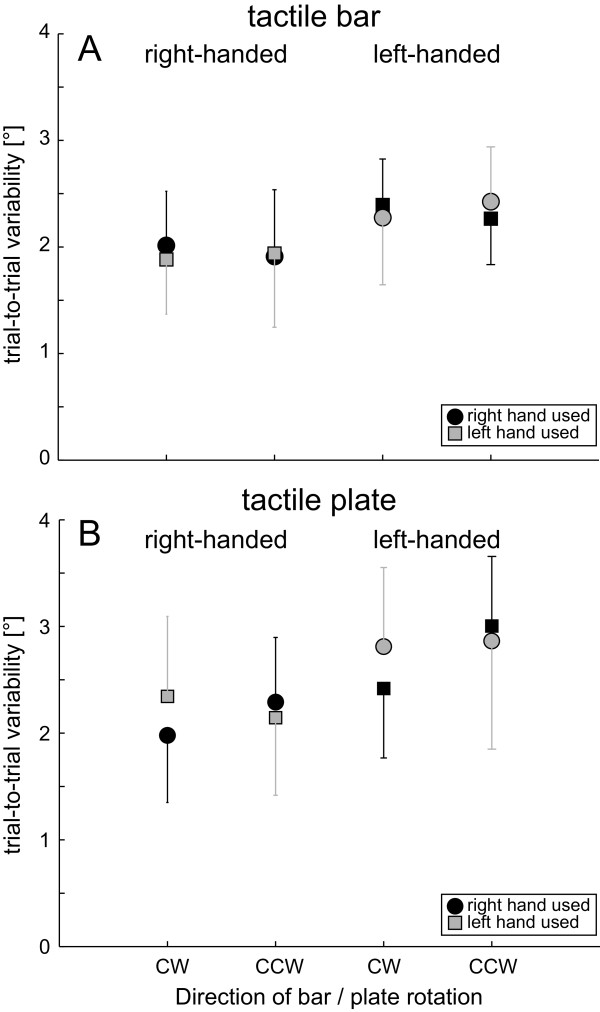
**Here average (± 1 StdDev) trial-to-trial variability of SHV is presented; results when using the tactile rod (panel A) and the tactile plate (panel B) are shown separately.** For description of the symbols and trial conditions see legend of Figure 3.

SHV variability in experiment 1 (Figure 4) was on average 2.3° (± 0.7°; ± 1 StdDev; pooling all conditions) and was found to be independent from the direction of rod/plate rotation (F[1,22] = 0.58, p = 0.447) and the hand used (F[1,22] = 0.33, p = 0.565) (5-way ANOVA). However, SHV variability depended on the type of grip (F[1,22] = 13.80, p < 0.001), the gender (F[1,22] = 7.20, p = 0.008) and handedness (F[1,22] = 32.71, p < 0.001): significantly larger trial-to-trial variability was noted when using the precision grip (plate) compared to the wrap grip (rod) (2.4 ± 0.8° vs. 2.1 ± 0.6°, p < 0.001), for left-handed subjects compared to right-handed subjects (2.6 ± 0.7° vs. 2.1 ± 0.6°, p < 0.001) and for female subjects compared to male subjects (2.4 ± 0.6° vs. 2.1 ± 0.7°, p = 0.003). Furthermore, a significant interaction between the gender and handedness (F[1,22] = 8.88, p = 0.003) for SHV variability was found, with pairwise comparisons showing significantly smaller average trial-to-trial variability for right-handed male participants relative to either right- or left-handed females and left-handed males.

### Perceived straight-ahead (experiment 2)

**Figure 5:**
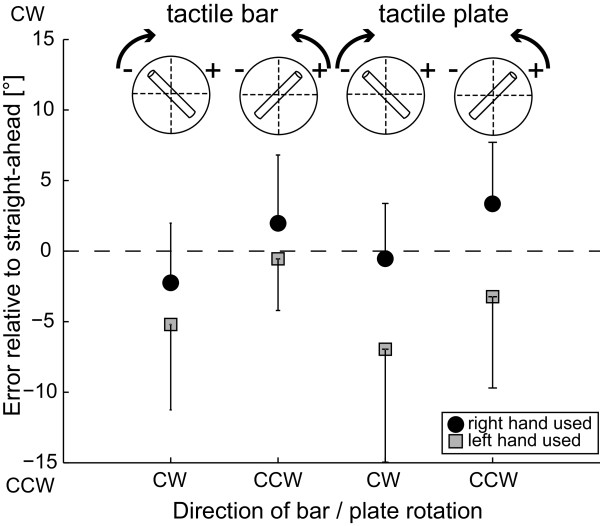
**Average (± 1 StdDev) errors of PSA (perceived straight-ahead) for both the tactile plate and the tactile rod in 10 right-handed subjects are shown.** For description of the symbols and trial conditions see legend of Figure 3.

Average errors of PSA relative to objective midline are shown in Figure 5. Note that in experiment 2 only right-handed subjects participated (for details see methods section). Overall, no significant (*t*-test, p > 0.05) errors were noted when pooling all PSA trials (irrespective of the hand used, the direction of rod/plate rotation, the type of grip and gender) (−1.7 ± 6.1°; average ± 1 StdDev). We found a tendency of each hand to deviate the Velcro-marked pole of the device towards its own hemispace. These errors relative to objective midline were significant (based on t-tests) only when subjects used their left hand (−4.0 ± 6.5°, p < 0.001), but not when they used their right hand (0.6 ± 4.7°, p > 0.05). Statistical analysis (4-way ANOVA) of PSA deviations showed a significant main effect for the hand used, yielding larger leftward deviations, as seen from the top, when using the left hand (F[1,18] = 14.57, p < 0.001). Furthermore a significant main effect for the direction of rotation of the tactile device with regard to the Velcro-marked pole (rightward rotations yielded significantly larger leftward errors than leftward rotations, F[1,18] = 11.64, p = 0.001) was noted, whereas the type of grip (F[1,18] = 0.08, p = 0.779) and the gender (F[1,18] = 0.26, p = 0.612) did not have an effect on PSA errors. No significant interactions between the type of grip, the direction of rotation of the tactile device and the hand used were observed.

**Figure 6:**
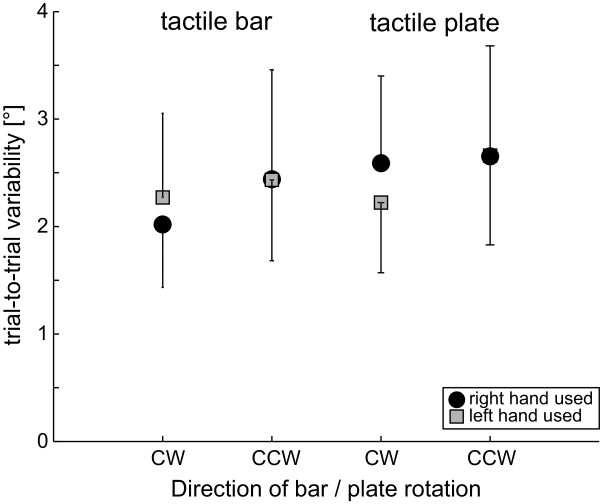
**Average (± 1 StdDev) trial-to-trial variability of PSA in right-handed subjects is presented; results when using the tactile rod and the tactile plate, respectively, are shown separately.** For description of the symbols and trial conditions see legend of Figure 3.

Overall PSA trial-to-trial variability averaged at 2.4° (± 0.8°, ± 1 StdDev) as indicated in Figure 6 and was independent of the gender (F[1,18] = 0.08, p = 0.777), the direction of rod/plate rotation (F[1,18] = 2.17, p = 0.145), the hand used (F[1,18] = 0.68, p = 0.412) and the type of grip (F[1,18] = 1.70, p = 0.196) (4-way ANOVA). No significant interactions (ANOVA) were noted.

### Synopsis of results of the two experiments

Pooling all SHV and all PSA trials, respectively, a small but significant average CCW deviation was noted for the SHV, but not for the PSA. For both the SHV and the PSA effects of the hand used and the direction of rod/plate rotation were found on adjustment errors. The latter effect was similar for SHV and PSA leading to significantly larger CCW shifts (relative to true earth-vertical and true straight-ahead, respectively) for CW rotations compared to CCW rotations. The effect of hand used, however, was opposite in the two experiments: while the SHV showed a CCW bias when the right hand was used and no bias for the left hand, in the PSA a CCW bias was obtained for the left hand without a bias for the right hand. No effects of grip (medium wrap vs. precision), gender and handedness (in SHV) on accuracy were observed, however, precision of SHV was significantly better in right-handed subjects compared to left-handed subjects, in males compared to females and when using the wrap grip instead of the precision grip. On average, the precision of adjustments was as good along the PSA as along the SHV.

## Discussion

Two experiments assessed different planes of action and different frames of reference, either focusing on an earth-fixed frame (gravitational vertical) in experiment 1 or on a body-fixed frame (body-midline axis) in experiment 2. Both revealed a tendency of adjustments to deviate counter-clockwise (CCW), i.e. top of the tactile device moving leftward in the roll plane in experiment 1 and tactile device pointing leftward in the horizontal plane in experiment 2. These shifts of either subjective haptic vertical (SHV) or perceived straight-ahead (PSA) relative to true earth-vertical and true straight-ahead, respectively, depended on the hand used and reached statistical significance (p < 0.05) only for adjustments using the right hand in experiment 1 and for adjustments using the left hand in experiment 2.

As in our study (investigating unimanual adjustments), others have previously noted offsets of perceived haptic vertical of several degrees in various whole-body roll-tilt positions for both unimanual [11,27] and bimanual [11] tasks. On the other hand, however, some studies have reported accurate bimanual rod adjustments along earth-horizontal [9] and earth-vertical [31] as well as accurate unimanual rod adjustments along earth-vertical [32,33]. Thus, the errors found in our unimanual experiments cannot be explained just by the fact that we used a unimanual task. Other factors potentially explaining these discrepancies in the size of adjustment errors between different studies are discussed below.

### Motor scanning direction-dependent differences in both the roll and horizontal plane

In both experiments CW rod/plate rotations led to significantly larger CCW shifts. We hypothesize that the direction-dependent differences in SHV and PSA adjustments reflect *hysteresis*, a property of systems whose states depend on their immediate history (Merriam Webster definition). Hysteresis accounts for the finding that the SHV and PSA are not unequivocally determined by the internal estimate of the gravitational vertical and the body midline, but depend on the previous history of the manipulating hand. In other words, the adjusted rod/plate orientation is biased towards the direction from which the SHV/PSA was reached. Lejeune and colleagues previously reported significant undershooting (up to ~6°) of adjusted rod orientation relative to the principle axes, proposing systematic errors in perceived haptic vertical and horizontal [10] and an effect of the direction of rod rotation, consistent with hysteresis. Studying the haptic vertical in various whole-body roll positions using a wrap grip, we [27] previously also noted hysteresis in right-handed subjects using their dominant hand. The observations made here and those from Lejeune [10] and Schuler [27], however, are in contrast to a study by Kerkhoff [32], where no effects of the direction of unimanual rod rotations in the roll plane in right-handed subjects were reported. Most likely, differences in the experimental paradigms used contributed to these discrepancies: Small sample size (Kerkhoff collected only 5 trials with CW and CCW rotation in each subject while 24 trials each were obtained in the study discussed here) might be such a parameter. On the other hand, the type of grip seems not to influence the emergence of hysteresis, as slight touch of the finger tips led to hysteresis in one study [10], while this was not the case in another study [32]).

A relevant gravitational contribution to the hysteresis observed here seems unlikely, as hysteresis of similar size and direction was found in planes both parallel (experiment 1) and perpendicular (experiment 2) to the gravity vector in our study. Also the differences in wrist and elbow joint receptor stimulation due to the distinct arm/hand positions to solve the two tasks did not crucially affect hysteresis. Different mechanisms may result in hysteresis. In visual processing short-term adaptation leads to changes in the orientation selectivity in the primary visual cortex [34,35], in vestibular stimulation the signal from the canals outlasts the stimulation through velocity storage [36] and may bias subsequent vestibular-driven movements. In analogy, short-term adaptation due to motor learning [37] could bias estimates of hand roll orientation towards the previously felt position of the hand. Short-term adaptation leading to hysteresis may therefore account for the finding that the SHV and the PSA are not only determined by the internal estimate of the gravitational vertical and body midline, but depend also on the previous motor history of the hand used.

### Effects of handedness and hand used

In both the roll plane and the horizontal plane the hand used had an effect on the size and direction (CW vs. CCW) of the adjustment errors. The effect of the hand used, however, was opposite in experiments 1 and 2, indicating that haptic tasks in these two planes may differ in certain properties. Whereas larger CCW errors were observed when using the *right* hand in the roll plane, larger CCW shifts were found when using the *left* hand in the horizontal plane. For both planes, a pattern resembling the one we observed here, has been reported previously by others: Studying unimanual adjustments along the SHV, Bauermeister et al. [11] described larger CCW deviations when using the right hand compared to the left hand, which matches our findings in the roll plane. Previous studies in the horizontal plane confirm our observation that adjustments with the left hand result in further CCW deviations than adjustments with the right hand [38-40]. However, we failed to reproduce significant CW deviations when using the right hand to indicate PSA as previously observed by others [39]. The reason for opposite effects of the hand used depending on the plane of action remains unclear. Possibly they are related to the specific arm position (stimulating the joint receptors and muscle spindles differently), its orientation relative to gravity, and the movement required for completion of the task. The fact that previous studies have reported the (qualitatively) same effects of right- vs. left-hand use for both planes of action supports the hypothesis that the SHV and the PSA task differ substantially in certain aspects and makes an effect restricted to the paradigms used in the experiments reported here unlikely. While for the SHV task grasping the rod/plate leads to extension in the wrist, the PSA task results in flexion in the wrist. Applying the rod/plate rotations with the wrist either flexed or extended might contribute to the switch of relative errors for adjustments with the right vs. the left hand in the two planes. However, we did not quantify the amount of wrist flexion/extension in the two experiments. This hypothesis, therefore, needs to be addressed with more rigorous measures in future studies.

We found SHV accuracy, i.e. the degree of veracity, in left- and right-handed subjects to be very similar and failed to show any significant differences as one might expect from theoretical considerations about differences in hemispheric activation, as left-handed subjects are thought to have less strongly lateralized hemispheric spatial functions [41,42]. Either such differences in hemispheric activation in right- and left-handed subjects are restricted to the horizontal plane-which seems rather unlikely - or the experimental paradigm used here did not induce such differences as both the right and left hemifield were stimulated to a similar degree. Whereas in the roll plane no previous study comparing adjustments in left- and right-handed subjects has been published to our knowledge, several studies have addressed this question in the horizontal plane. Scarisbrick and colleagues [40] reported larger CCW deviations of the dominant hand in visual line bisection for left-handed subjects compared to right-handed subjects. These findings were later reproduced by Luh and colleagues [43], who also found larger CCW deviations in visual line bisection for left-handed subjects. As Luh and colleagues [43] studied the dominant hand in right- and left-handed subjects only, the differences may possibly be related rather to the hand used than to handedness. Our data-though obtained in the roll plane - would support this hypothesis as deviations of the dominant hand in right-handed subjects roughly matched deviations of the non-dominant hand in left-handed subjects and vice versa. Scarisbrick and colleagues [40] showed that adjustments with the right hand were very similar in both left- and right-handed subjects. For the left hand, however, larger CCW deviations were noted in left-handed subjects, which is different from what we observed in the roll plane.

### The precision of SHV is affected by the handedness, the gender and the type of grip

Our data suggest that unimanual adjustments of the SHV and the PSA when upright allow precise estimates of perceived vertical and straight-ahead with trial-to-trial variabilities ranging between 2 and 2.5°, similar to the range observed for the subjective visual vertical (range: 1.0 – 3.0° [44-48]). The finding that left-handed subjects were less precise in adjusting the SHV than right-handed subjects was unanticipated. Probably the strength of lateralization had an influence on the task precision, yielding larger trial-to-trial variability in case of a weaker hemispheric lateralization. This hypothesis is supported by the observation that left-handed subjects are less strongly lateralized in terms of hemispheric spatial functions [41,42]. Based on the existing literature that suggests superiority of the non-dominant hand in utilizing proprioceptive input [23-26], we would have expected differences in precision related to the hand used (yielding superior precision for the non-dominant hand), rather than related to the handedness. An effect of the hand used (dominant vs. non-dominant hand) on precision, however, was observed neither in the roll nor in the horizontal plane. The finding that males were significantly more precise in adjusting the SHV than females deserves further attention. Gender effects on the precision of the SHV have not been addressed in previous studies to our knowledge. Whether such a gender effect is indeed characteristic for the haptic vertical needs to be addressed in future studies.

Several issues may contribute to the observed increase in trial-to-trial variability for the precision grip applied to the rectangular plate in experiment 1. On one hand, the number of joints involved in the roll movement is increased when adjusting the plate as it depends both on the wrist and the joints of the fingers, whereas adjustments of the rod mainly relied on a single joint, the wrist. Thereby the degrees of freedom for the precision grip are increased, resulting in more sources of noise. On the other hand, tactile input is limited to the fingertips in the precision grip, whereas the entire palm is stimulated in the wrap grip. This restriction may impair estimates of the device’s roll orientation further. However, the density of tactile sensors at the fingertips is higher than the average density on the entire palm. Whether the varying distribution of tactile sensors stimulated in the two tasks resulted in distinct skin proprioceptive signal-to-noise levels or not, therefore remains open. Furthermore, in contrast to the study by Lejeune et al. [10], the accuracy when using the precision grip compared to the wrap grip was not inferior. In contrast to experiment 1, increases in variability with the precision grip in the horizontal plane (experiment 2) did not reach statistical significance (p > 0.05). This could be interpreted either as a relatively smaller contribution of tactile input to precise pointing straight-ahead than to precise pointing along perceived vertical or as an effect of the smaller sample size in experiment 2 compared to experiment 1 (10 vs. 21 subjects).

### Can the CCW shifts and direction-dependent effects be explained by pseudo-neglect?

In the literature, haptic adjustments along perceived vertical while being in an upright position have been found to vary substantially. Reported SHV alignments ranged from being very accurate [32] to showing systematic errors up to ~5° into CCW direction [11,49]. Leftward deviations of the PSA have been linked to the spatial nature of the task, which may preferentially activate the right hemisphere [50-53] with resultant enhancement of the left perceptual field [40,54]. In the horizontal plane, leftward deviations were previously reported for pointing straight-ahead [15,38,39] and for visual and tactile [13-15] line bisection tasks, a phenomenon referred to as “pseudo-neglect” [12]. The discrepancies between the overall non-significant leftward deviations noted here when pointing straight-ahead and the significant leftward deviations found in a meta-analysis [15] with a larger study group (n = 156) are possibly related to the small sample size (n = 10) used here. Whereas pseudo-neglect may explain leftward deviations observed in the horizontal plane, we will further elaborate on the hemispheric activation pattern of the paradigms used here in the paragraph below and show that the hemispheric activation theory can neither explain the pattern of deviations in the roll plane nor the scanning-direction effects observed in either the roll or the horizontal plane.

The theoretical considerations that have led previous authors to propose an asymmetric hemispheric activation to explain scanning direction effects in PSA depend on the assumption that the tactile stimulus is represented preferentially in one hemifield. For the rod/plate used here, however, this is not the case. The tactile device was anchored in the mid-sagittal plane and the axis of rotation was in the center of the device for both SHV and PSA. Subjects were instructed to hold the device in the center of rotation. Therefore touching the rod while rotating it will always stimulate both perceptual hemifields, e.g. for PSA a rightward rotation will move the more distant half of the device into the right hemifield and the nearer half into the left perceptual hemifield. Short-term adaptation leading to hysteresis (as discussed further above) may be sufficient to explain the motor-scanning direction-dependent differences without postulating asymmetric hemispheric activation.

The pattern of SHV deviations found here is contrary to what would be expected based on the activation-orientation theory of pseudo-neglect and both our data and previous studies addressing pseudo-neglect in the horizontal plane: left hand use in the horizontal plane was associated with larger CCW deviations in the study reported here. In the roll plane, however, we observed the opposite: left hand use was associated with significantly smaller CCW deviations than right hand use, despite the predicted stronger right-hemispheric activation (resulting in a larger CCW shift) by left hand use according to [55]. One potential interpretation of these findings would be that pseudo-neglect in tactile-spatial tasks is limited to certain planes, i.e. the horizontal plane in our study. A restriction of pseudo-neglect to the horizontal plane, however, seems rather unlikely, as the stimulus will fall into the right and left tactile hemifield in a similar way in both planes of action. The underlying mechanisms for the SHV pattern found in the roll plane (smaller CCW deviations when using the left hand instead of the right hand) therefore are still not convincingly explained.

Another argument that the hysteresis noted here is of motor and not of pseudo-neglect origin is that we found the same hysteresis for haptic alignments in both the horizontal and the roll plane. While for the horizontal plane the argument of pseudo-neglect can be discussed, there is no indication in the roll plane that the upper half of an object moving into one hemifield (e.g. into CW direction) has a higher impact as the lower half moving in the other hemifield (e.g. into CCW direction) when positioned in the frontal plane straight-ahead.

## Conclusions

We found systematic, direction-dependent deviations of the subjective haptic vertical, which could not be attributed to gender, handedness, the hand or the kind of grip used. These deviations depended on the starting position and the direction of rotation of the haptic device, reflecting hysteresis, and were independent of the direction of gravity as they could be reproduced in a pointing straight-ahead task. We propose that short-term adaptation, shifting the attention towards previous adjustment positions, may provide an explanation for such biases in spatial orientation in both the horizontal and frontal plane. Overall we noted no evidence for pseudo-neglect in the roll plane and found only little support for a relevant role of pseudo-neglect in our pointing straight-ahead task. The influence of the previously postulated right-hemispheric dominance for spatial performance on haptic alignment tasks therefore remains controversial.

When precise SHV adjustments are crucial, both the amount of tactile and kinesthetic input should be maximized and the degrees of freedom of joints involved in the movement should be minimized. When considering the use of a rod to assess perceived vertical and perceived straight-ahead, one has to be aware of slight CCW shifts in either plane (roll plane: top to left; horizontal plane: pointing direction to the left), which may reach statistical significance. Furthermore, one has to control for the hand used to minimize trial-to-trial variability values, as significantly different deviations relative to earth-vertical and relative to straight-ahead were noted for adjustments applied with the right vs. left hand.

## Competing interests

The authors declare that they have no competing interests.

## Authors’ contributions

AAT conceived of the study, performed the experiments and the statistical analysis and participated in drafting the manuscript. JS performed the experiments. CJB participated in the study design and its coordination. DS helped formulating the study hypotheses and drafting the manuscript. All authors read and approved the final manuscript.
